# Comparative gene-expression profiling of the large cell variant of gastrointestinal marginal-zone B-cell lymphoma

**DOI:** 10.1038/s41598-017-05116-3

**Published:** 2017-07-20

**Authors:** Thomas F. E. Barth, Johann M. Kraus, Ludwig Lausser, Lucia Flossbach, Lukas Schulte, Karlheinz Holzmann, Hans A. Kestler, Peter Möller

**Affiliations:** 1 Institute of Pathology, Ulm University, Ulm, Germany; 2 Institute of Medical Systems Biology, Ulm University, Ulm, Germany; 3 Core Unit Genomics, Ulm University, Ulm, Germany

## Abstract

Gastrointestinal (g.i.) large cell lymphoma is currently regarded as diffuse large B-cell lymphoma (DLBCL) despite a more favorable clinical outcome compared to other DLBCL. Cluster analyses on a transcriptome signature of NF-κB target genes of 30 g.i. marginal zone B-cell lymphomas (MZBL; 8 g.i. MZBL, 22 large cell MZBL - among them 9 with coexisting small cell component) and 6 DLBCL (3 activated B-cell like (ABC), 3 germinal center-like (GCB)) reveals a distinct pattern. The distinctiveness of large cell MZBL samples is further confirmed by a cohort of 270 available B-cell lymphoma and B-cell *in silico* profiles. Of the NF-κB genes analyzed, *c-REL* was overexpressed in g.i. MZBL. *c-REL* amplification was limited to 6/22 large cell MZBL including the large cell component of 2/9 composite small cell/large cell lymphomas, and c-Rel protein expression was found in the large cell compartment of composite lymphomas. Classification experiments on DLBCL and large cell MZBL profiles support the concept that the large cell MZBL is a distinct type of B-cell lymphoma.

## Introduction

Marginal zone B-cell lymphoma (MZBL) of the mucosa-associated lymphoid tissue (MALT) is an indolent neoplasm with a typical small cell morphology^1^. Moreover, in the g.i. tract, a more aggressive, large cell variant (large cell MZBL) exists and composite lymphomas (ComL) occur, containing both distinct small and large cell components. According to the current WHO classification^2^, these large cell lymphomas should be diagnosed as diffuse large B-cell lymphoma (DLBCL) either with or without residing MZBL component. There is growing evidence that these g.i. large cell MZBL are clinically less aggressive than the typical DLBCL^3–7^. We showed previously that the morphologically different parts of ComL are clonally related, and demonstrated that the large cell parts are blastically transformed g.i. tract MZBL^8–11^. In this follow-up study, we now investigate whether the pathogenesis of g.i. large cell MZBL translates into an expression signature distinct from those of other DLBCL.

Extranodal marginal zone B-cell lymphomas are known for their of NF-κB dysregulation^12^. Therefore, we narrowed the cluster analysis to a set of NF-κB target genes shown to be highly discriminative between the two major DLBCL subtypes, germinal center (GCB) and activated B-cell like (ABC) subtype^13–15^. Hence, we compared gene expression profiles of g.i. large cell MZBL with and without small cell components with those of 270 B-cell lymphoma entities such as Burkitt’s lymphoma (BL), DLBCL, primary mediastinal B-cell lymphoma (PMBL), pulmonary and g.i. MZBL lymphoma, follicular lymphoma (FL), and mantle cell lymphoma (MCL), and of non-neoplastic B-cell populations. Furthermore, the NF-κB target gene signature was used to conduct a classification analysis of the DLBCL and large cell MZBL profiles. Relabelling experiments are used to prove identified groups. Therefore, such experiments were performed in order to analyze whether or not MZBL is a learnable and discriminable subtype of DLBCLs besides the activated B-cell like (ABC) and the germinal center B-cell like (GCB)^13–15^.

From these experiments, we conclude that large cell MZBL is a separate group distinctive of conventional DLBCL.

## Results

### Cluster analyses

The Ulm cohort was analyzed in an agglomerative hierarchical cluster analysis. The corresponding results are given in Supplemental Figure S1, panel A. The dendrogram revealed three main clusters which essentially coincide with: A) DLBCL of GCB and ABC subtypes, B) large cell MZBL, and C) g.i. MZBL lymphoma. The common branch between large cell MZBL and g.i. MZBL from the dendrogram also supports our previous findings that the large cell parts are blastically transformed g.i. tract MZBL^8–11^.

**Figure 1:**
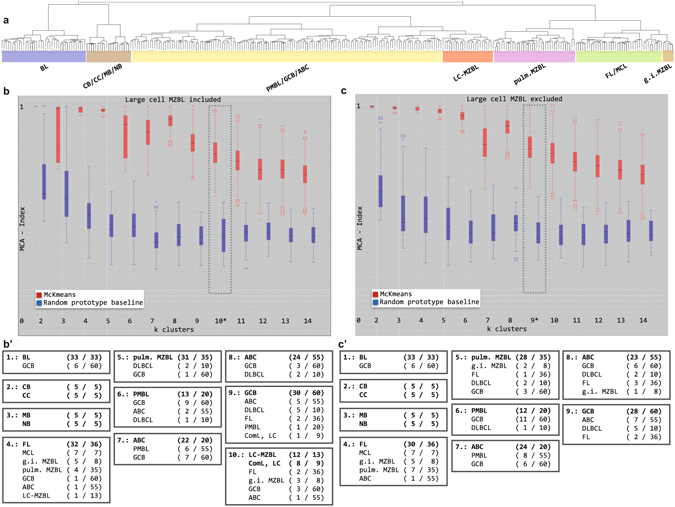
Comparison of partitioning cluster analyses with and without the large cell variant of MZBL based on the NF-κB target genes (n = 271). Upper panel (a) Agglomerative hierarchical clustering indicates a separate LC MZBL cluster. Middle panel: cluster number estimation using the k-means algorithm ((**b**) large cell MZBL included) and ((**c**) large cell MZBL excluded). Most stable results are found for ten clusters (**b**) and nine clusters (**c**), respectively. Lower panel: composition of the ten clusters (b’; large cell MZBL included) and the nine clusters (c’; large cell MZBL excluded).

We performed cluster analysis experiments on the basis of a NF-κB target gene signature. These genes were previously published to be highly discriminative between the two major DLBCL subtypes^16^. Hierarchical clustering utilizing the NF-κB target genes on the Ulm cohort also coincided with the whole genome cluster results (Supplemental Figure S1, panel B). The initial cohort was extended by 270 profiles of Burkitt’s lymphoma (BL), nodal DLBCL, primary mediastinal B-cell lymphoma (PMBL), follicular lymphoma (FL) and mantle cell lymphoma (MCL), pulmonary MALT lymphoma, and normal B-cell populations. Figure 1, panel a shows an overview of the extended cohort as an agglomerative hierarchical clustering. To estimate the correct number of clusters, which best reflects the underlying data structure, we performed resampling experiments^17^. Figure 1, panel b provides an overview on these resampling experiments. For each cluster number k a box plot of the cluster validation index (MCA-index) for the k-means clustering (red) and random clustering (blue) is shown. A high value of the MCA-index corresponds to a robust partitioning that is not affected by resampling. The highest k (k = 10) with a significant difference of the MCA-index corresponds to the most stable cluster solution. In panel b’ this partitioning is shown. This clustering essentially coincides with branches of the hierarchical clustering shown in panel a^17^. These clusters comprised basically the following groups: 1. BL, 2. centrocytes and centroblasts, 3. memory B-cells and naïve B-cells, 4. FL, MCL, and g.i. MZBL lymphoma 5. pulmonary MZBL, 6. PMBL and GCB, 7. PMBL and ABC, 8. ABC, 9. GCB, and 10. large cell MZBL comprising also ComL, LC (Figure 1,b’). Furthermore, FL, ABC, and GCB lymphomas from the Ulm cohort cluster with the corresponding groups from the whole cohort, which confirms that the composition of the cohort is unbiased. For a discussion of possible batch effects see also Supplementary Figures S6–S9. To prove the independence of the resampling cluster analysis from the MZBL profiles the analysis was repeated excluding the large cell MZBL. The composition of the nine most stable groups (Figure 1,c) was compared in Figure 1, panels b’ (large cell MZBL included) and c’ (large cell MZBL excluded). The profiles in the corresponding groups were almost the same, supporting the independence of the cluster groups from the large cell MZBL profiles and the presence of a distinct large cell MZBL category.

### Classification analyses

We performed classification experiments on the NF-κB target gene signature in order to investigate whether the large cell MZBL can be seen as an independent category of DLBCLs. We utilize the linear support vector machine (SVM) as classification method. SVM takes into account all genes of a signature and does not include an additional feature selection process^18^.

A classification model is trained to distinguish the concepts of large cell MZBL, ABC and GCB and therefore receives a subset of samples. The learnability of the classes can be estimated by the model’s accuracy in predicting the class labels of the remaining samples. We compared the learnability of the original classes to other hypothetical concepts, which were generated by relabeling the available samples (e.g., by exchanging the class label of a large cell MZBL sample and an ABC sample; for details see supplemental text on classification analyses).

**Figure 2:**
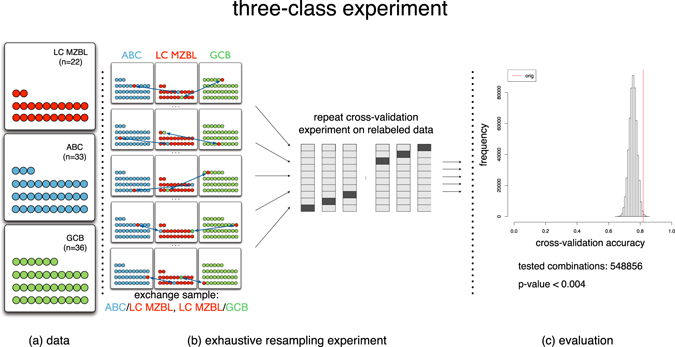
Three-class experiment based on the signature of 271 NF-κB target genes. Negative control: sensitivity of random group re-assignment. Panel (a) shows a pictogram of the datasets. The learnability of the concepts ABC, GCB and large cell MZBL is estimated in a 10 × 10 cross-validation experiment (quality measure: accuracy, linear support vector machine). Panel (b): The accuracy of the original experiment is compared to accuracies gained on perturbed datasets. Here, two samples of the large cell MZBL class are exchanged with one sample of the ABC and one sample of the GCB class. This experiment is repeated for all possible combinations of data points (n = 548856). Panel (c): Results of the cross-validation experiments. Histogram of the accuracies achieved. The red bar indicates the accuracy achieved in the original experiment (p < 0.004). For a higher number of label exchanges than two, lower average accuracies were achieved (data not shown). This means that for both approaches the results are highly reproducible.

The results of the supervised classification experiments support that the large cell MZBL are not a group of arbitrarily chosen samples (Figure 2). For the three-class data set consisting of ABC, GCB, and large cell MZBL samples, an accuracy of 82% was achieved which further strengthens this hypothesis. Relabeling the samples caused a degeneration of the classification performance. A better or equal accuracy could only be achieved by chance for p < 0.004 of all relabeled data sets, making this result statistically significant. Including the large cell MZBL samples in the category of ABC or GCB samples showed a similar effect, i.e. for these two class experiments only p < 0.007 of all data sets allowed a better or equal classification than for the original set of ABC/GCB samples (Supplemental Figure S3). These results strengthen the hypothesis that large cell MZBL is a standalone and learnable class.

### Gene expression signature

Additionally, we investigated the presence of a large cell MZBL specific gene expression signature. Within the set of NF-κB target genes we identified 24 differentially expressed genes (log2 fold change >1; shrinkage T-test, FDR < 0.05) between large cell MZBL and ABC, large cell MZBL and GCB, respectively. This gene expression signature is summarized in Table 1 and Supplemental Figure S4. It can be categorized into genes referring to extracellular space, plasma membrane, and protein binding (Gene Ontology categories, Fisher exact test, FDR < 0.05).

**Table 1:** A gene expression signature separating large cell MZBL from ABC and GBC classes. The 24 differentially expressed genes (log2 fold change > 1; shrinkage T-test, FDR < 0.05) can be categorized in genes referring to extracellular space, plasma membrane, and protein binding (Gene Ontology categories).

Category	HUGO	ABC vs MZBL	GCB vs MZBL
Extracellular space			
	CD36	1.08	0.92
	CXCL13	0.96	1.69
	EBI3	0.35	1.17
	IL1B	0.97	1.29
	CXCL8	1.73	1.82
Plasma membrane			
	ACKR3	1.63	0.85
	FAS	1.78	2.23
	PTPN1	0.15	1.11
	RASGRP1	0.43	1.03
	RGS1	1.39	1.27
Protein binding			
	AHR	0.91	1.34
	BCL2A1	0.32	1.01
	BIRC3	0.97	1.17
	CNTRL	1.05	0.91
	EGR1	1.01	0.66
	MAP3K1	0.76	1.14
	MAP3K8	1.12	1.21
	PRPF4B	0.99	1.42
	REL	1.16	0.79
	SOD2	0.99	1.18
Other			
	BANK1	1.42	1.95
	ELL2	0.49	1.14
	FNDC3A	0.88	1.11
	PTGS2	1.75	1.64

### *c-REL* gene and c-Rel protein analysis

One of the overexpressed genes is the proto-oncogene *c-REL*. We therefore analyzed this gene by FISH with *c-REL* specific probes as well as on the protein level (Supplemental Table S3 and Figure S5). We found that *c-REL* copies in terms of high-level amplifications were limited to 6/18 large cell MZBL samples. In contrast, 8 g.i. MZBL samples showed a diploid state of *c-REL*. In two composite lymphomas, the amplification was detected in the large cell component only. One ABC DLBCL sample was *c-REL* amplified. For protein analysis we first performed transfection experiments with HEK cells to prove the specificity of the staining of the antibody used (Supplementary Figure S5E,F). Staining of the lymphoma cells revealed 4 different patterns: negative, positive in the cytoplasm, positive in the nucleus, and positive in the cytoplasm and the nucleus (Supplementary Figure S5A–C). The intensity of the staining was compared to strong positive staining of germinal centers of tonsillar tissue (Figure S5D). The 7 *c-REL* amplified lymphomas all showed a nuclear and a cytoplasmic c-Rel staining. However, the nuclear and cytoplasmic staining pattern was not limited to the amplified lymphomas but also detected in 28 lymphomas without *c-REL* amplification (3 only nuclear; 7 only cytoplasmic; 18 cytoplasmic and nuclear; see Supplementary Table S3).

## Discussion

We show in a transcriptomic approach that in a small cohort of B-cell lymphoma, DLBCL, large cell MZBL, and g.i. MZBL are distinct groups. We also show that by comparison with a large cohort of 270 lymphomas and B cell subtypes, generally speaking, almost every B-cell lymphoma entity described in the WHO^2^ corresponds to one group based on 271 NF-κB target genes, thus substantiating a discrimination of these B-cell lymphomas at the transcriptional level. This analysis revealed that large cell MZBL is its own group different from DLBCL.

In the analyses, BL samples were clearly separated from the other B-cell lymphoma entities. This separation may result from a generally low level expression of NF-κB target genes shown by expression profiling analyses^19^. Aiming at identifying specific pathway activation patterns in DLBCL, Bentink *et al*. found a distinct and unique pattern for BL, illustrating again the outstanding position of this lymphoma entity^20^.

Analysis of the DLBCL revealed 5 clusters by partitional clustering based on NF-κB target genes. These clusters consisted of a ABC and GCB group as well as PMBL split with ABC and GCB clusters. Given the fact that NF-κB activation is only one of several discriminating factors between PMBL and DLBCL^21, 22^, it is reasonable that a clustering based solely on NF-κB target genes is responsible for this result. In line with this finding is that by comparison of PMBL profiles with ABC DLBCL and their newly characterized “host response” DLBCL subtype, Feuerhake *et al*. found both common and distinguishing features concerning NF-κB target gene expression^15^ accounting for heterogeneity within the DLBCL entity. Furthermore, Monti *et al*. described three consensus clusters in DLBCL without any correlation to the previously defined subtypes (GCB, ABC or “other”)^23^. In addition, Bentink *et al*. have published four recurrent pathway activation patterns in DLBCL, which were also completely unrelated to the subtype (GCB or ABC) or to the consensus clusters defined by Monti *et al*.^20^.

The fifth DLBCL group detected consists of the large cell MZBL containing the large cell component of the composite g.i. lymphomas (ComL, LC). This result defines the large cell MZBL as category distinct from DLBCL. The profiles of three of our g.i. MALT lymphomas (MALT 1, 4 and 8) were also assigned to this group by the different approaches used. Reevaluating the histomorphology of these lymphomas, they contained up to 20% blastic cells, being a morphological hallmark of malignant transformation that was reflected by the cluster analyses.

To exclude possible discriminative effects solely based on nodal *vs*. extranodal location, we screened the affiliation files of the DLBCL analyzed for samples originating from a primary extranodal location. There were only a few extranodal samples (at least two, according to the affiliation files from Pasqualucci^16^) and they were distributed all over the different DLBCL groups. Hence, the results are not biased by the primary location of the lymphoma.

The supervised classification approach additionally showed that large cell MZBL is a learnable concept. In particular, the two-class experiment revealed that a large cell MZBL sample cannot be assimilated within the classes of ABC or GCB in a learning scenario. The three class experiment additionally underlines that large cell MZBL is a learnable class label and therefore a distinct group. Compared to ABC DLBCL and GCB DLBCL we identified a large cell MZBL gene signature of 24 differentially expressed genes, which were categorized in genes referring to extracellular space, plasma membrane, and protein binding. From this signature we have analyzed in detail the genomic, transcriptional and protein level of *c-REL* since gastro-intestinal B cell lymphomas are an example of general activation of the NF-κB pathway^24, 25^. *c-REL* amplifications in DLBCL from the ABC/GCB type are found in up to 15%; therefore this gene does not differentiate the large MZBL from other DLBCL on the genomic level^26^. This finding was confirmed since 1 DLBCL of 6 analyzed revealed a *c-REL* amplification.

However, we found that amplification of this gene was detected only in the large cell MZBL while the small cell compartment and small cell MZBL showed no genomic amplification of this proto-oncogene pointing to a role of this gene during lymphoma progression. c-*REL* amplification did not necessarily correlate with nuclear c-Rel protein expression. Similar findings have been described in DLBCL with co-amplification of several genes, which also map to 2p^27^. These genes, involved in processes such as deubiquitination and degradation of IkB, are found to be highly co-expressed in *c-REL* amplified cases^27^.

**Figure 3:**
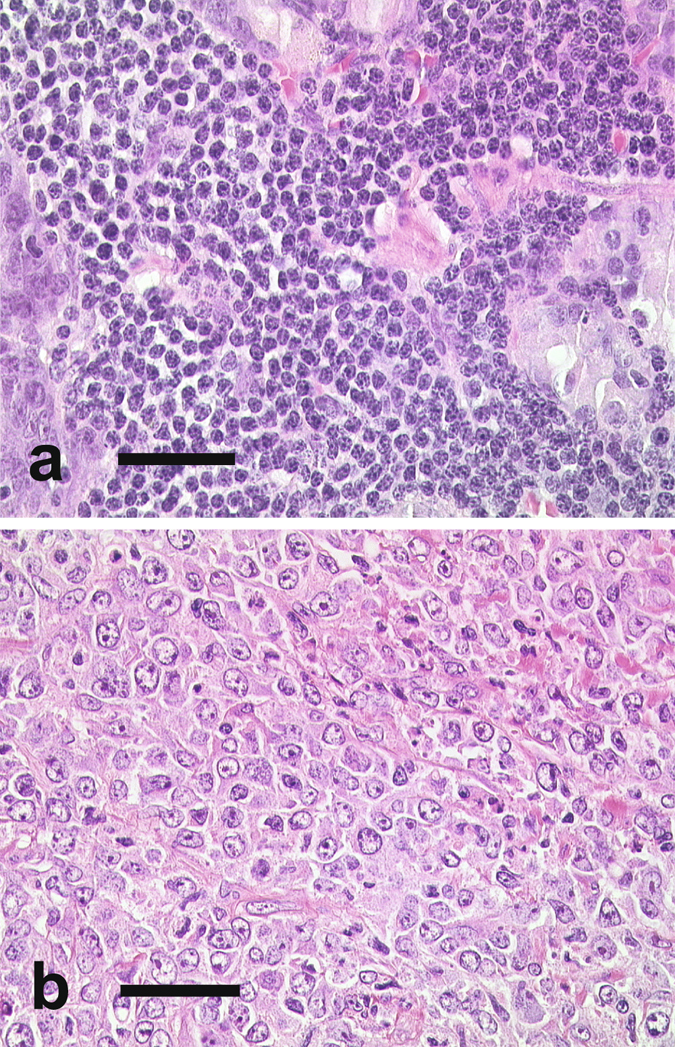
Histology of a typical small cell gastric marginal zone B-cell lymphoma (**a**) and large cell variant of a gastric marginal zone B-cell lymphoma with blastic cytology ((**b**); bar = 50 μm).

To summarize, our comparative analyses support the view that the large cell MZBL (shown paradigmatically in Figure 3 in comparison to a g.i. small cell MZBL) is a distinct category and thereby confirm biological, cytogenetic, and clinical data that these lymphomas are distinct from hitherto defined DLBCL.

## Methods

### Patient samples

Frozen tissue samples of two FL and 30 primary g.i. MZBL were taken from the tissue collection of the Institute of Pathology, Ulm University, Germany. The lymphomas were pseudonymized to comply with the German law for correct usage of archival tissue for clinical research^28^. All the performed methods and experiments were in line with the guidelines of the ethics committee of the Federal General Medical Council. The research was carried out in compliance with the Helsinki Declaration. All experimental protocols were conducted and approved in accordance with the local ethics committee of the University of Ulm (usage of archived human material 03/2014). In essence, the MZBL panel used here is close to identical with the formerly published lymphoma series, which has been characterized by SNP profiling, molecular cytogenetics, and gene expression profiling^9, 10, 29, 30^ (see Supplemental Table S2 for a case identifier list). This study included eight small cell MZBL (g.i. tract MZBL lymphomas), thirteen large cell MZBL, and nine large cell areas of composite lymphomas (ComLlarge cell). We further included 6 nodal diffuse large B-cell lymphomas 3 of GCB and 3 of ABC type each characterized by immunophenotyping using antibodies for CD10, BCL6, and MUM1 as described^31^. All investigated samples contained at least 90% of tumor cells.

### Fluorescence *In Situ* Hybridization

To determine the *c-REL* genomic status, we performed fluorescence *in situ* hybridization (FISH) using a REL gene-specific probe and a chromosome 2 centromeric probe as described^32^.

### Immunochemistry

A polyclonal anti-cRel antiserum (Cell Signaling, p/n 4727) was used in a concentration of 1:100 on paraffin section of 2–4 µm. To evaluate the specificity of the serum transfection experiments with HEK293 cells were performed using human embryonic kidney cells 293. These cells were transiently transfected with the Rc/CMV2 vector and the Rc/CMV2 plasmid with the full-length *REL* cDNA according to the manufacturer’s protocol (Fugene; Roche Biochemicals, Mannheim, Germany). Cells were pelleted and fixed in formalin 48 hours after transfection and embedded in paraffin. Subsequently 2 to 4-μm thick paraffin sections of the cell pellets were subjected to immunocytological staining (see “Immunohistochemistry”). As detection system we used the EnVision Kit (Dako, Carpintera, CA) according to standard protocols described elsewhere^33^. Evaluation of immunostaining was carried out in a blinded fashion on a multihead microscope by two of us (TFEB; LS). In tissue sections, strongly stained immunoblasts or lymph follicles were regarded as positive intrinsic controls. Several categories were created according to the proportion of cells displaying positive staining for c-Rel: − stands for no staining detected, + indicates staining in up to 30%, ++ indicates staining in more than 30% and up to 70%, and +++ indicates staining in more than 70% of the total number of cells analyzed (Table S3)^33^.

### Array data analysis

The chips were scanned with an AffymetrixGeneChip Scanner 3000 and subsequent images analyzed using GCOS 1.4 (Affymetrix). Arrays have been normalized using the robust multiple-array average method (RMA)^34^. Array data are available at Gene Expression Omnibus (GEO), accession ID GSE39577: http://www.ncbi.nlm.nih.gov/geo/query/acc.cgi?token = trklfkocqeswwxy&acc = GSE39577.

### Other published datasets used

Additional datasets created with the HG U133Plus 2.0 chip were obtained from the GEO database. We used gene expression profiles from 119 DLBCL^16, 19^ (GSE12195), 20 PMBL^19^, 33 BL^19^ (http://llmpp.nih.gov/BL), 36 FL^16, 35^ (GSE16024 and GSE12195), 7 MCL^35^ (GSE16024), 35 pulmonary MALT lymphomas^36^ (GSE13314) and 20 purified B-cell populations^16^ (centroblasts, centrocytes, naïve B-cells and memory B-cells, each five samples, respectively; GSE12195). Information about the DLBCL subtype (ABC, GCB) was obtained from the corresponding affiliation files. For an overview of the used data sets see Supplemental Figure S2.

### Cluster analyses

We first performed agglomerative hierarchical cluster analysis on the whole genome data. To compare samples from the different cohorts, a subset of NF-κB target genes^16^ (represented by approximately 300 probes) was selected (for a complete list see Supplemental Table S1). To estimate the optimal number of clusters a resampling based robust partitioning cluster algorithm (McKmeans) was applied and compared to a random prototype baseline^17, 37^.

### Classification analyses

For further validation of the results we performed a supervised analysis limited to DLBCL and large cell MZBL based on the NF-κB target gene signature (for further details see supplemental methods). Relabeling experiments were conducted in order to investigate whether large cell MZBL can be seen as an independent subtype of DLBCL. All classification experiments were conducted with help of the TunePareto Software^38^.

## Electronic supplementary material

Supplementary Information

Supplementary Table 1

Supplementary Table 2

Supplementary Table 3
