# Supplementary material for: Comparative gene-expression profiling of the large cell variant of gastrointestinal marginal-zone B-cell lymphoma
Source: Sci Rep. 2017 Jul 20;7:5963. doi: 10.1038/s41598-017-05116-3 (PMC5519735; doi:10.1038/s41598-017-05116-3)
Supplement: Supplementary file 1 — Supplementary Information [file 41598_2017_5116_MOESM1_ESM.pdf]

Supplemental data

to

Comparative gene-expression profiling of the large cell variant of gastrointestinal marginal-zone B-cell lymphoma

Thomas F.E. Barth[1+], Johann M. Kraus[2+], Ludwig Lausser[2+], Lucia Flossbach[1*], Lukas Schulte[1], Karlheinz Holzmann[3], Hans A. Kestler[2*+#], Peter Möller[1+#]

[1]Institute of Pathology; [2]Institute of Medical Systems Biology, [3]Core Unit Genomics; Ulm University, Ulm, Germany

*Corresponding author; [+]contributed equally,[#]shared last authorship

Supplementary Figures and Tables

Figure S1

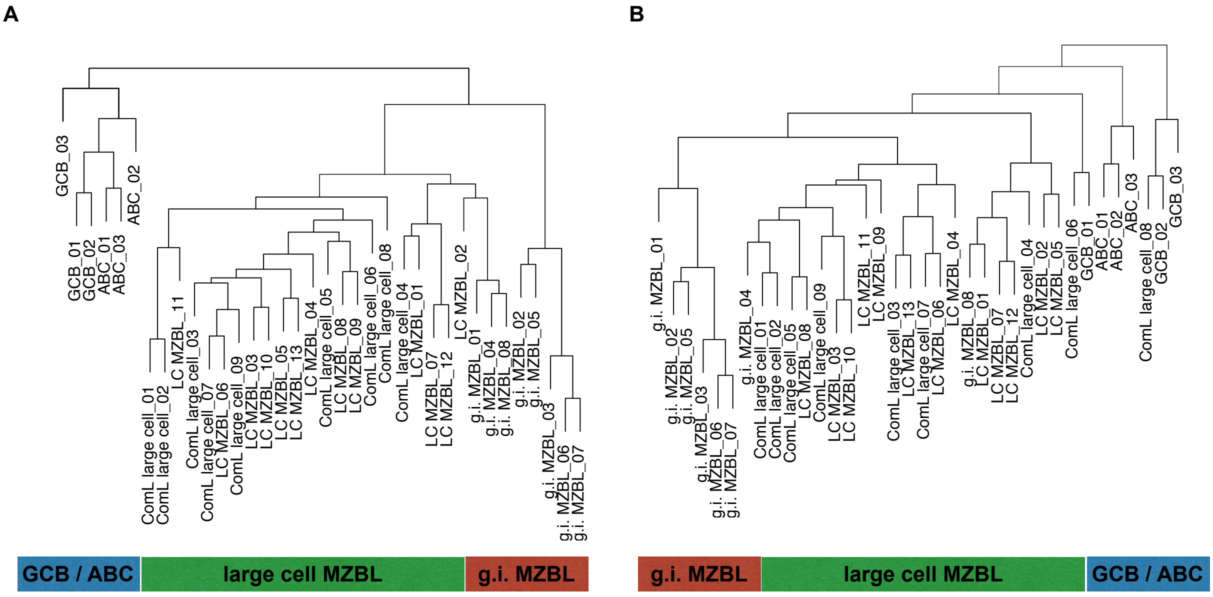

Figure S1: Agglomerative hierarchical clustering of the small B-cell lymphoma cohort on whole genome signature (A) and restricted to the NF-κB target genes (B).

Figure S2

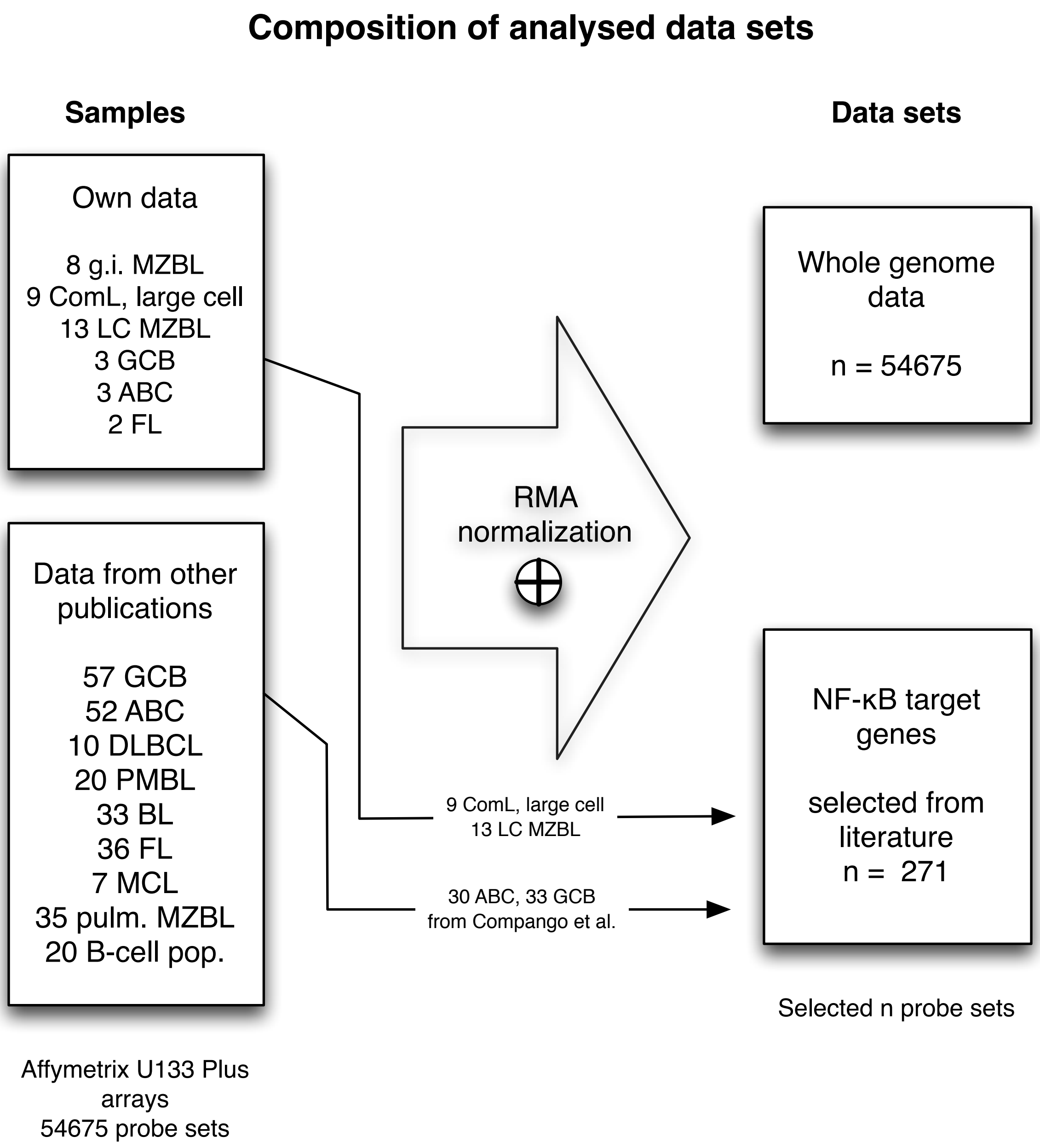

Figure S2: Composition of the data sets used. All data set were normalized with robust multiple-array average (RMA) normalization (Gautier et al. Bioinformatics, 2004).

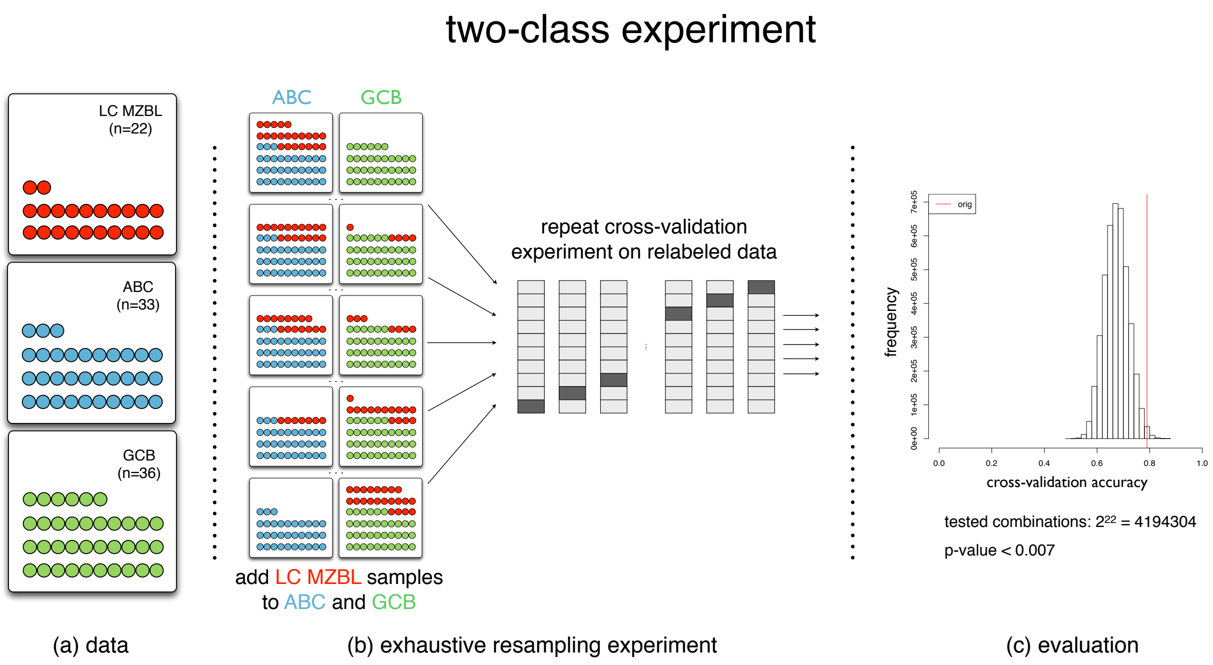

Figure S3: Two-class experiment based on the signature of 271 NF-κB target genes.

Negative control: categorization under assumption that there are only two classes (ABC and GCB) present in the data. The figure shows the influence of the LC MZBL samples on the learnability of ABC and GCB. Panel (a) shows a pictogram of the datasets. The learnability of the concepts ABC and GCB is estimated in a 10 x 10 cross-validation experiment (quality measure: accuracy, linear support vector machine). Panel (b): The influence of the large cell MZBL class is estimated by utilizing these samples as additional training data for ABC and GC. The experiment is repeated for all possible ways of splitting the large cell MZBL samples (n= 4194304). Panel (c): Results of the cross-validation experiments. Histogram of the accuracies achieved. The red bar indicates the accuracy achieved by assuming three distinct classes in the data ($p<0.007$).

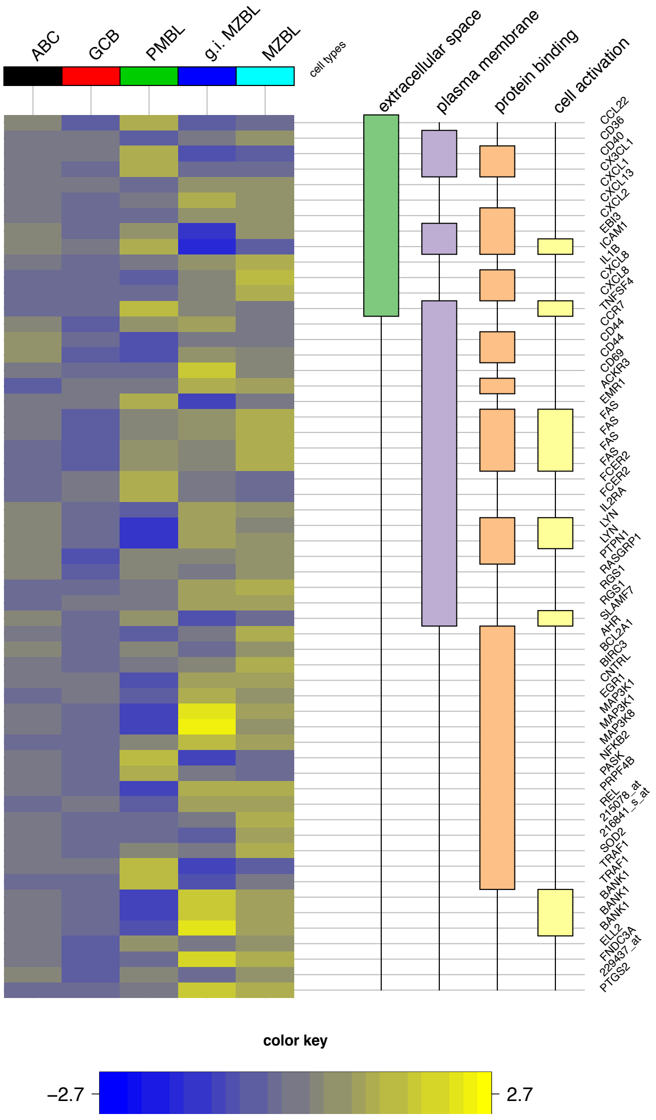

Figure S4: Gene expression signature of the large cell MZBL entity compared to ABC/GCB DLBCL, PMBL, and g.i. MZBL.

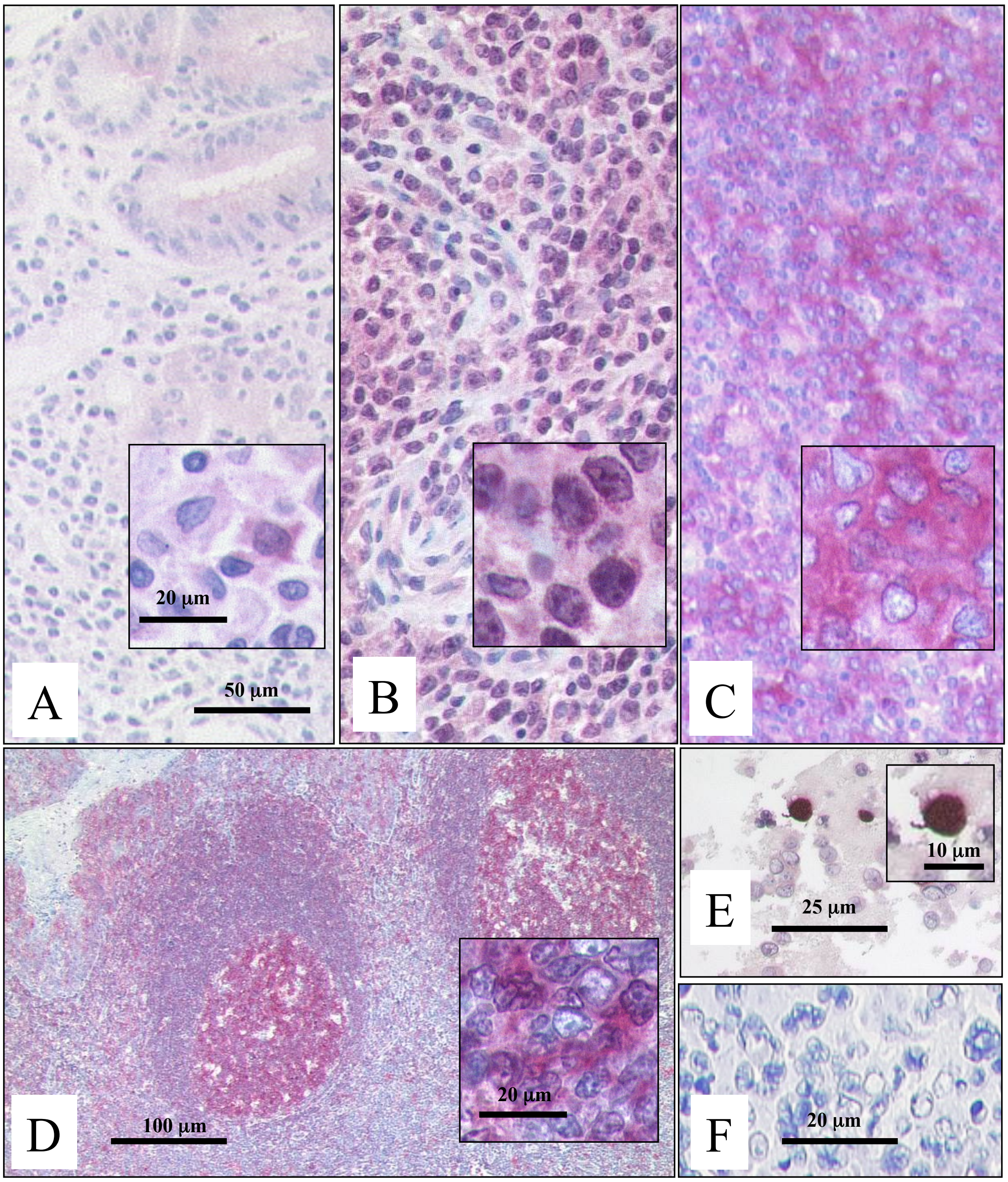

Figure S5: Immunhistochemical staining of c-Rel protein. A) Small cell marginal zone B-cell lymphoma of the stomach (giMZBL_02; diploid *c-REL* status); the lymphoma cells are negative for c-Rel protein; a immunoblast is weakly c-Rel-positive. B: Large cell g.i. B-cell

lymphoma (LCMZBL_06; diploid *c-REL* status); a weak nuclear expression of c-Rel protein is seen in all nuclei; a faint cytoplasmic staining is detected in some of the cells. C: Large cell g.i. B-cell lymphoma; LCMZBL_05; amplified *c-REL* gene); c-Rel protein is expressed in the cytoplasm and in some of the nuclei of the lymphoma. Tonsil with c-Rel staining: Germinal centers are strongly positive; the marginalzone is weakly positive; some immunoblasts in the extafollicuar space are c-Rel-positive; insert shows cells from the germinal center with nuclear and cytoplasmic staining. E) Transfected HEK cells are strongly positive for c-Rel protein; F) negative control; HEK cells.

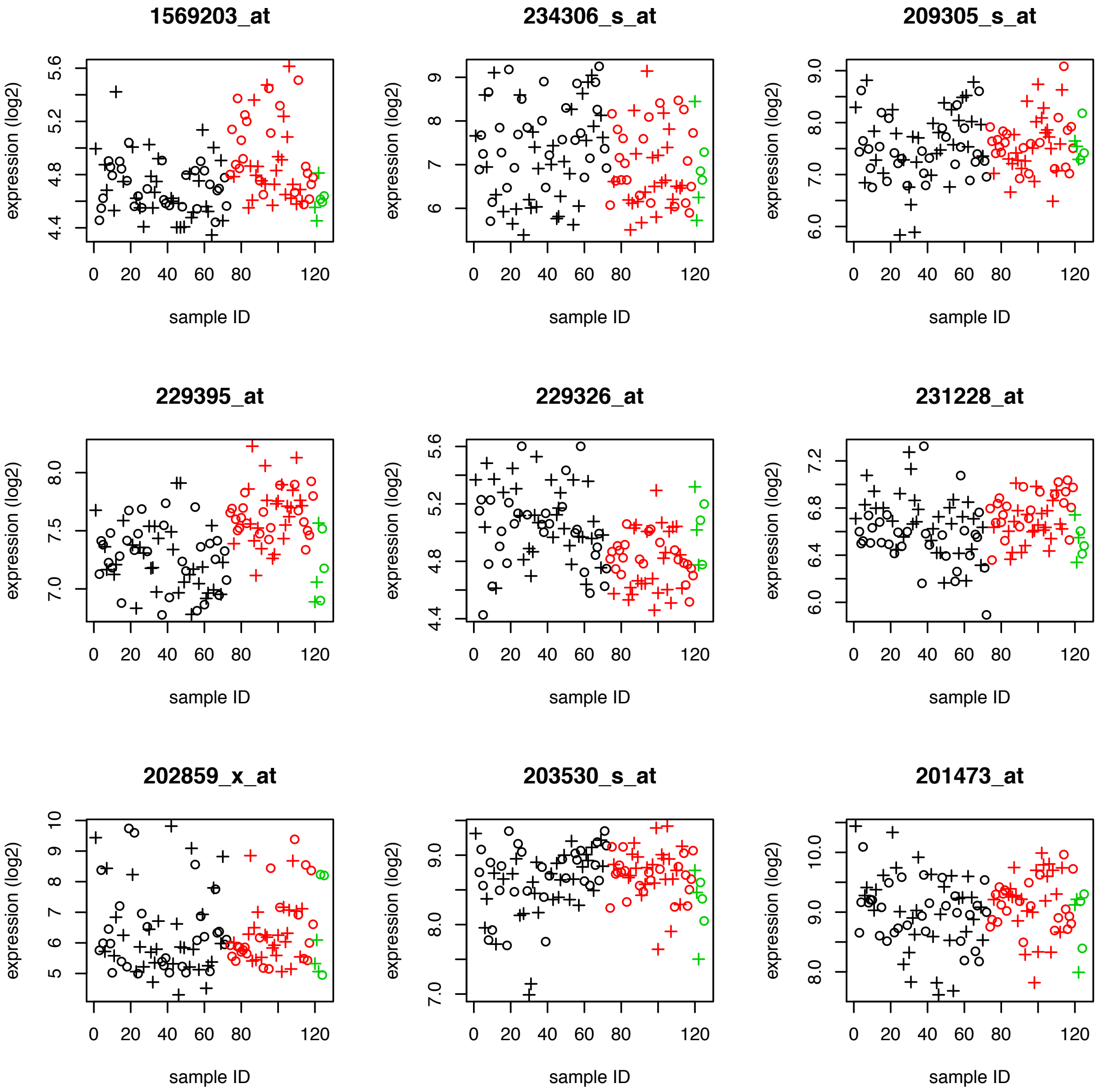

Figure S6: Log2 expression values from ABC/GCB samples (+, o) for 9 randomly selected genes. Colors (black, red, green) represent three different batches, i.e., Compagno[15], Dave[17], and Ulm cohort. There is no shift in expression correlating with batches or tumor subtype.

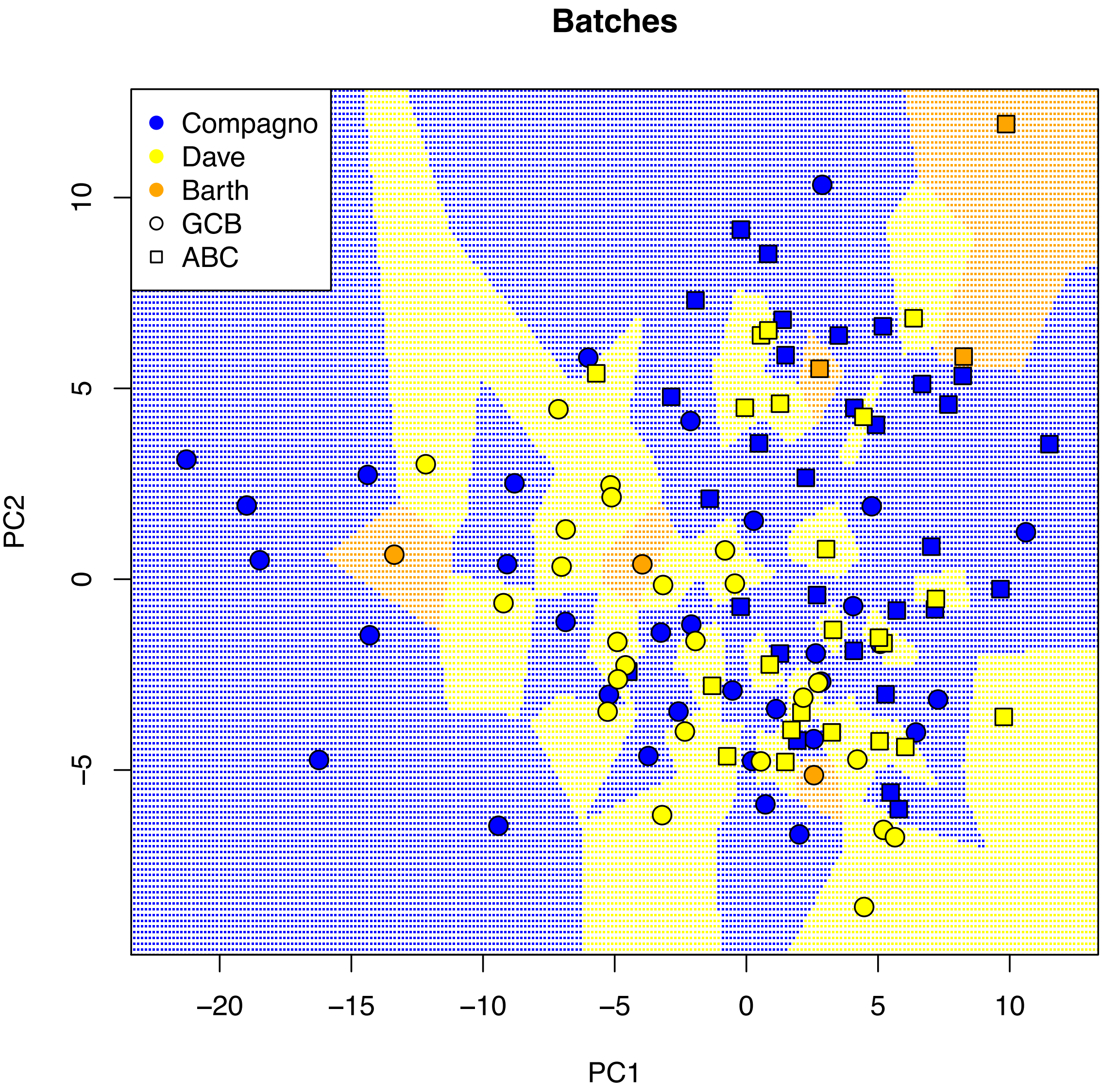

Figure S7: Projection of the samples onto first two principal components labelled by batch. Samples from different batches are shattered.

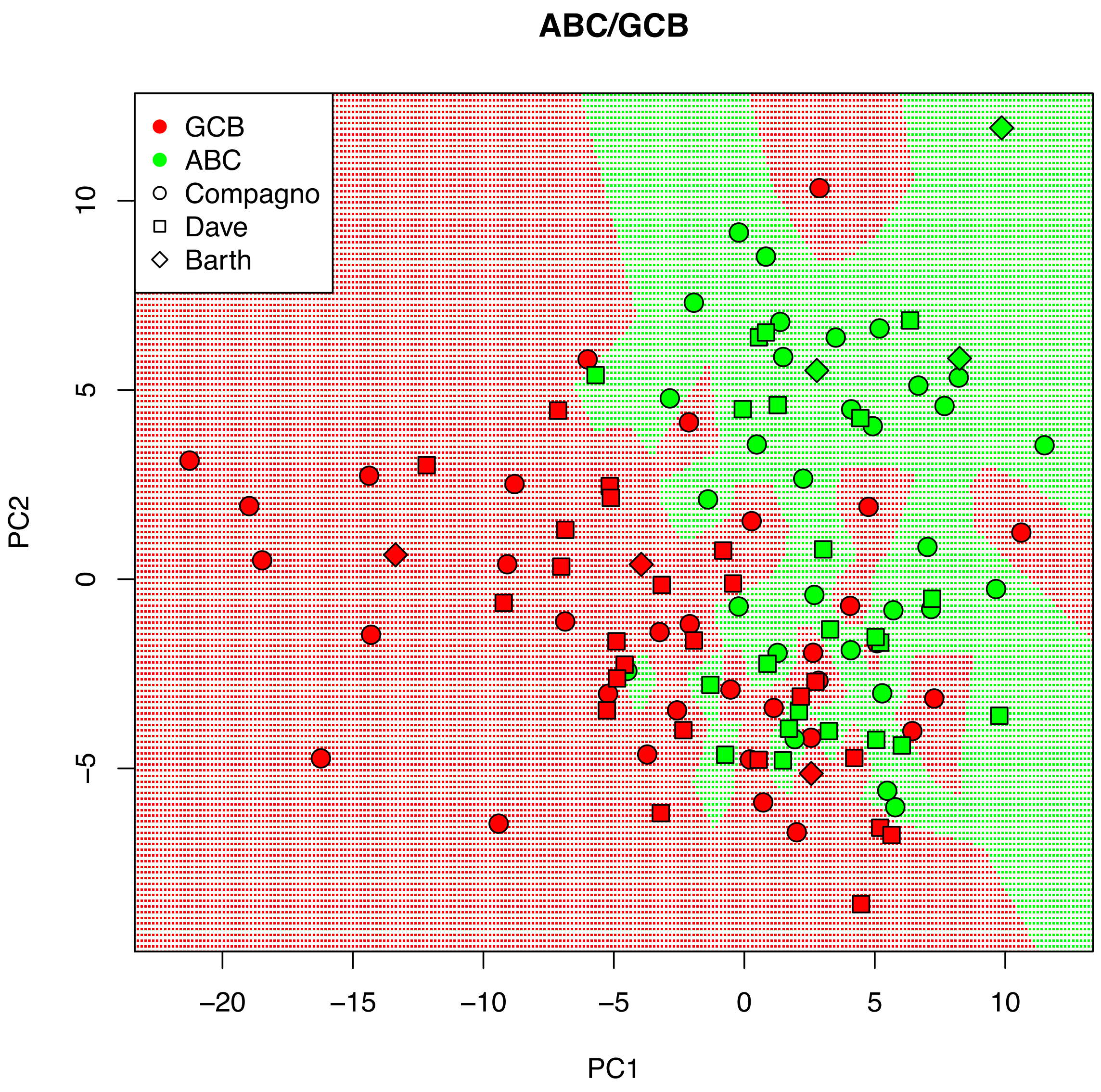

Figure S8: Projection of the samples onto first two principal components labelled by tumor subtype. The samples group well according to the tumor types.

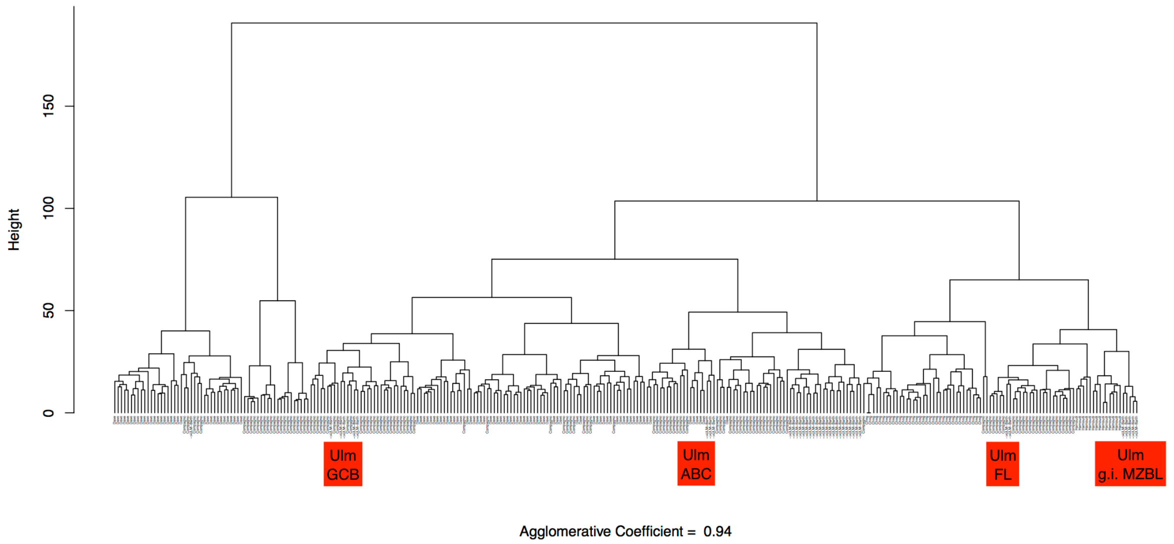

Figure S9: Hierarchical clustering of normalized data annotated by batch labels. Samples from same tumor subtypes cluster together, although they originate from different batches, e.g., ABC, GCB, FL from Ulm cohort. For a comparison to tumor subtypes see also Figure 1A.

Table S1: List of NF-κB target genes and their representation on the Affymetrix Chip used in our analyses.

Table S2: Case identifier of the lymphoma samples used by us in this and preceding publications.

Table S3: Correlation of c-Rel protein expression and REL genomic status.

Classification analyses and setup

The task of a classification analysis is to train a mathematical model, a so-called classifier that allows predicting the category (e.g., the lymphoma type) of an object according to a set of measurements (here, gene expression levels). In an initial phase the classifier is adapted (trained) according to a set of samples for which the correct categories are already known. The training of a classifier can be seen as a learning process for the concepts of the analyzed

categories. If it is learned correctly, it will allow an accurate prediction of the categories of new unseen samples. The accuracy is usually estimated on an independent set of test samples. In our experiments a linear support vector machine (SVM) was utilized as classifier.

Both phases, training and testing, require a certain amount of samples to achieve both a good adaptation and a stable estimation of the prediction accuracy. If the number of available samples is limited, training and testing can be done in a more sophisticated (resampling) experiment, which allows for optimally utilizing the data for both purposes. An example for such a resampling experiment is the 10 x 10 cross-validation. With this approach a dataset is split into 10 folds of about equal size (Figures 2b and S3b). A classifier is trained on 9 of these folds (white boxes, Figures 2b and S3b). The tenth fold is used for testing the accuracy of the classifier. The procedure is repeated until each fold has been tested once. The predicted labels of the test samples are compared to the corresponding true labels. The rate of correct prediction is used as an estimate for the accuracy of the classifier. This procedure is repeated on 10 randomized versions of the dataset.

For our classification analyses we combined the DLBCL data of Compagno *et al.*(Compagno *et al*, 2009a) with our own dataset, consisting of 22 large cell MZBL, 33 ABC and 36 GCB samples. Two different experimental setups based on a two-class and a three-class experiment were performed on the signature of 271 NF-κB target genes (Figure S3). 10x10 cross-validation results were compared between the original dataset and on slightly mislabeled (perturbed) versions of the data (Figure 2b and S3b). Linear support vector machines were used as classification models (Müssel *et al*, 2012).

One setup was concerned with the categorization into the two classes ABC and GCB (supplemental Figure S3). A classifier trained on the original data was compared to classifiers trained on slightly mislabeled datasets. The 22 large cell MZBL samples were relabeled as ABC or GCB for this experiment. The prediction accuracies were determined for all possible

combinations (2^{22} = 4194304). They were compared to the accuracies achieved on original ABC and GCB samples (see results).

Relabeling results

For relabelling experiments we first investigated how the large cell MZBL samples influenced the learnability of the concepts of ABC and GCB (Figure 2 and S3). The accuracy achieved in the experiments on the original ABC and GCB cells was compared to the experiments for which the single large cell MZBL cells were assigned to be either ABC or GCB samples (Figure S3). As a base experiment a 10 x 10 cross-validation experiment was chosen. The relabeled datasets were exhaustively analyzed for all possible relabelings of the large cell MZBL cells (2^{22} combinations). The accuracies achieved for these new hypothetical concepts can be summarized in a histogram (Figure S3c). Compared to the accuracy achieved on the original ABC/GCB dataset it emerged that only 0.6% of all possible relabelings achieved a better performance. This means that in 99.4% of all experiments the large cell MZBL samples diminish the prediction accuracy of ABC and GCB. In a second experiment we investigated if large cell MZBL can be seen as a separate concept (Figure 2). Here the basic task is to predict accurately the category of ABC, GCB, and large cell MZBL samples. Again the performance achieved on the original categorization is compared to the performance on slightly relabelled datasets (Figure 2b). The labels were perturbed by exchanging the class label of two large cell MZBL samples with the class labels of an ABC sample and a GCB sample. The experiment was again repeated for all possible relabelings (n=548856). Figure 2C shows that only 0.4% of all combinations achieve a better accuracy. This means that approximately 3 of a thousand relabeled datasets can be learned better than the categorization into ABC, GCB, and large cell MZBL.

Batch effect correction

Our protocol for handling batch effects is based on Leek et al., Nature Review Genetics 11:733-739, 2010:

1. Additional hybridization of DLBCL, g.i. MZBL and FL samples from Ulm

To investigate a possible batch effect, we also included 6 DLBCL (3 ABC / 3 GCB), 8 g.i. MZBL, and 2 FL cases from Ulm. This enables the visual inspection of batch effects via clustering.

2. Batch normalization via RMA

Normalization adjusts global properties of measurements for individual samples to enhance comparability within a group of samples. Normalization (e.g., quantile normalization) does not adjust for between-batch differences when combining normalized batches of data (Johnson et al., Biostatistics 8(1):118-127, 2007). Therefore, we normalized all data combined into a single batch using robust multi-array average (RMA). When raw CEL-files from Affymetrix microarray are available, this procedure has been shown to outperform methods which process data in separate batches, e.g., fRMA (McCall et al., Biostatistics 11(2):242-253, 2010).

3. Hierarchical cluster analysis annotated by batches

To check for possible batch effects still present in the data we performed hierarchical clustering for all samples annotated by the different batches. Samples from two studies (Brune et al., JEM 205(10):2251; Fernandez et al., *Cancer Res* 2010;70:1408-1418) showed a significant batch effect, i.e., samples from these batches clustered in separate branches (data not shown). These data were processed using a different wet-lab protocol. As we were not able to remove this batch effect using fRMA, SVA or ComBat, this data was excluded in an early stage of the study.

4. Univariate analysis of random features via correlating log2 expression values to batch surrogates and tumor subgroups

Figure S6 shows log2 expression values for ABC/GCB samples (+, o) for 9 randomly selected genes. Colors represent three different batches, i.e. Compagno[15], Dave[17], and Ulm cohort. Furthermore, there is no significant batch effect for all NFkappaB genes (Anova, fdr<0.05).

5. Principal component analysis (PCA) to correct for components that correlate with batch surrogates

Principal component analysis was applied on DLBCL samples from Compagno, Dave and Ulm cohort to inspect batch and tumor classes. The first two principal components have 88% cumulative proportion of variance. Figure S7 and S8 show a projection of the samples onto these principal components either labelled by batch or by tumor subtype. While no separation can be seen for batches (S7) the samples group well according to the tumor types (S8). Background is colored according to Voronoi tessellation.

6. Reclustering of batch corrected samples

Figure S7 shows hierarchical clustering of all samples included in the study after batch correction. Samples from same tumor subtypes cluster together, although they originate from different batches (e.g., Ulm ABC/GCB/FL/g.i.MZBL marked in red). For a comparison to tumor subtypes see also Figure 1A.
